# Results of Posterior Dislocation of Elbow Associated with Bony and Soft Tissue Injury

**DOI:** 10.5704/MOJ.1303.010

**Published:** 2013-03

**Authors:** Neel M Bhavsar, Jyotish G Patel, Pankaj R Patel, Jigar B Chhapan

**Affiliations:** Department of Orthopaedics, SMT. NHL Municipal Medical College, Gujarat, India; Department of Orthopaedics, SMT. NHL Municipal Medical College, Gujarat, India; Department of Orthopaedics, SMT. NHL Municipal Medical College, Gujarat, India; Department of Orthopaedics, SMT. NHL Municipal Medical College, Gujarat, India

## Abstract

Elbow trauma is challenging to manage by virtue of its complex articular structure and capsuloligamentous and musculotendinous arrangements. We included 17 patients with elbow dislocation and associated injuries in this study. The study protocol included early elbow reduction and planned fixation of the medial or lateral condyle, coronoid and radial head. The sample was 73% male and 27% female with mean duration follow-up of 8 months, and mean age of 37 years. The mean Mayo Elbow Performance Score was 96 points at conclusion of follow-up, indicating an excellent result in 14 patients. Whenever the radial head was excised, we performed a strong transosseous ligamentous repair of the medial and lateral collateral ligaments. Fixation of the coronoid is essential for elbow stability. A small avulsed fragment can be fixed using an ACL jig. We found this technique very useful. Early planned intervention, stable fixation, and repair provide sufficient stability and enhance functional outcomes.

**Key Words:**

elbow dislocation, ligament instability, coronoid fracture

## Introduction

Trauma to the elbow can be challenging to treat by virtue of the complex articular structure as capsuloligamentous and musculotendinous arrangements. Improved understanding of elbow injuries has led to rapid evolution of treatment concepts. This elbow injury pattern is frequently associated with the disruption of the lateral band of the ulnar collateral ligament, as it is the first structure to be disrupted in the Horti circle of soft tissue disruption of the elbow, which refers to the three consecutive stages of lateral to medial progression of elbow dislocation^1^,^2^. The lateral band of the ulnar collateral ligament, disrupted in the first stage, is critical to elbow stability and its reconstruction may be crucial in the restoration of the joint^3^. Fracture of the coronoid associated with this injury is usually small and frequently involves the tip of the coronoid process^4^. Biomechanically speaking, a type I fracture of the coronoid involving just the tip is not a substantial insult to elbow stability, but a type II injury or worse significantly increases elbow instability^3^. Fixation of the more significant coronoid fractures may, therefore, be beneficial for the patient.

Ring et al reported that in a series of 11 patients with the terrible triad injury pattern, long-term results were unsatisfactory for the majority of patients 5. More recently Pugh et al. and Egol et al., concluded that these are difficult injuries and that even with optimal care, recurrent instability is possible; however, they can be treated using a standard surgical protocol including fixation of fractures and soft tissues^6^,^7^. Here, we present a retrospective study of patients with elbow instability.

## Materials and Methods

We identified 17 patients with irreducible elbow dislocation or elbow instability. Inclusion criteria consisted of the following: adult with acute closed fracture dislocation of elbow or with acute elbow instability. We excluded patients with open injury to the elbow, neglected elbow dislocation, patients with previous elbow surgery and patients treated conservatively. All patients had presented at the emergency department and were treated at our institution between February 2010 and December 2011. As this was a retrospective study and no new treatment was included to complete the study, our institution did not require approval by the research ethics board. Two patients were lost to follow-up prior to definitive assessment of the outcome, leaving fifteen patients for evaluation (11 males and 4 female with a mean age of 37y (range 8-53Y). Mechanisms of injury included falls, high-velocity falls from a height, bicycle accidents and motorcycle accident. We treated the fifteen elbows surgically at a mean of 4.5 days (range, 0-17d) after the injury. Specific indications for operative intervention included the following: a displaced periarticular fracture; an inability to obtain or maintain a concentric reduction following closed reduction; and residual instability of the elbow in a functional (30°- 130°) arc of motion following closed reduction. Radiographs taken at the time of presentation showed that all fifteen elbow dislocations were posterior.

Surgical treatment was based on the following algorithmic approach. Generally, we repair damaged structures sequentially from deep to superficial, using the lateral Kocher approach (coronoid to anterior capsule to radial head to lateral ligament complex to common extensor origin). Elbow stability was then evaluated, with the goal being concentric stability with no observed posterior or posterolateral subluxation through a flexion-extension arc of 20° to 130° with the forearm in neutral rotation. Instability was typically most evident in extension and supination. Lateral soft-tissue structures were disrupted and repaired in all patients. Patients with medial epicondyle or lateral epicondyle fracture were fixed with percutaneous wire or cannulated cancellous screw(s) and checked for instability.

Coronoid fractures were classified according to the Regan and Morrey classification system. A jig used for anterior cruciate ligament reconstruction (ACL tibial jig) was used to precisely fix these fractures. We positioned the jig such that one part of it caught the coronoid and the other was outside over the olecranon. We inserted wires from outside towards the coronoid, thus fixing the coronoid. If the fragment was very small, a non-absorbable suture was passed through the soft tissue attachment and fixed to the ulna. Detachment of the lateral ligament complex from the humerus was repaired with non-absorbable sutures placed through drill holes in the distal humerus. If unacceptable instability persisted, the medial collateral ligament was exposed and repaired. We used an ulnohumoral pin for additional stability only if grossly unstable elbows despite other fixation. Wounds were closed in layers, after which a sterile dressing was applied.

A well-padded posterior plaster splint was applied with the elbow in 90° of flexion and the forearm fully pronated to protect the lateral repair and maintain reduction. If both the medial and the lateral soft tissues were repaired, the forearm was splinted in neutral rotation. The ulnohumoral pin was removed at two weeks. Supervised motion was started within fifteen days of surgery, when the sutures and splint were removed; this included active and active-assisted range-of- motion exercises, including both flexion and extension. Flexion and extension exercises were performed with the forearm in pronation and active forearm rotation exercises with the elbow at 90°. We instructed patients to avoid the terminal 30° of extension until four weeks postoperatively, although most patients avoided this position anyway because of pain. Unrestricted shoulder and wrist motion was encouraged.

Patients were followed up every 15 days for 2 months postoperatively and then every 2 months. They were followed clinically and radiographically until fracture union occurred and elbow motion was regained with appropriate supervised physiotherapy. Clinical evaluation included determination of pain, function, range of motion, and stability. Anteroposterior and lateral radiographs were assessed for fracture union, implant loosening, heterotopic ossification, degenerative changes, and joint congruity. The Mayo Elbow Performance Score (MEPS) was determined for each patient at the final clinic visit 8.

## Results

**Figure 1:**
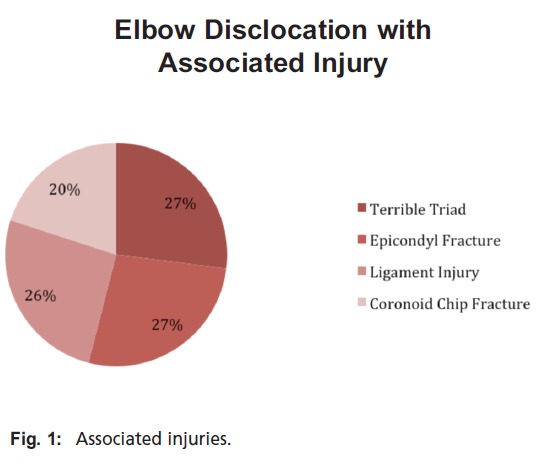
: Associated injuries.

Seventy-three percent of the 15 study patients were males and the mean duration of follow up was eight months (range 5-24m). The mean age was 37 years (53 to 80), and 66% of patients had injury to their dominant side. Mechanisms of injury included falls, high-velocity falls from a height, bicycle accidents and a motorcycle accident. Four patients presented with the terrible triad of elbow injury, two with coronoid fractures, two with medial and ulnar collateral ligament injuries, four with condylar fracture (either medial or lateral) and one with an isolated radial head with ligament instability (Figure 1). The mean arc of flexion extension was 1230 with mean flexion of 1320 .The functional arc of motion, as determined according to the criteria of Morrey et al. (a flexion-extension arc of 30°-130° and 100° of forearm rotation)1, was achieved in 14 out of 15 patients.

**Figure 2:**
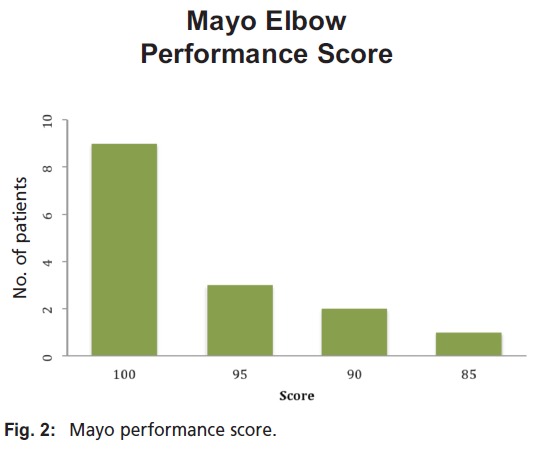
: Mayo performance score

At the time of follow-up, 14 of 15 patients maintained concentric reduction of both ulnotrochlear and radiocapitellar articulations. The mean Mayo Elbow Performance Score (MEPS) was 96 points (range, 45 to 100 points), corresponding to an excellent result in fourteen elbows and a good result in one (Figure 2).

All coronoid and radial head fractures treated with internal fixation had solid osseous union on final follow-up radiographs. Calcification in the medial and lateral ligaments was common, seen to some degree in 9 of the 15 elbows. Heterotopic ossification was evident in 2 patients, but neither required additional treatment.

Three patients (7%) developed complications. One patient developed radiographically confirmed valgus instability (valgus stress view). The second developed joint stiffness with decreased range of motion of elbow due to periarticular ossification, and a third patient complained of persistent severe pain during elbow motion and excessive activity. In contrast, we saw one radiograph in which there was excessive periarticular ossification, but that particular patient did not have any complaints and had full range of motion. There was no association between terrible triad injuries and subsequent complications; however complications were associated with advanced age and osteoporotic bone.

## Discussion

Acute instability of the elbow due to fracture dislocation is difficult to manage. In this short term study, a majority of the patients did well according to the functional Mayo elbow performance score. Most patients presented after high velocity injury or a fall suggesting the severity of force required to cause such injuries. Urgent primary care via reduction and planned secondary procedure is required for such injuries.

Egol et al.^6^ and Pugh et al. 7 conducted studies on the standard surgical protocols for the terrible triad of elbow. They reported mean Mayo scores of 81 and 88 points respectively; ours was slightly better, but this may be due to the fact that they included only patients with the terrible triad. Both studies suggested fixing all soft tissue and bony structures when possible and replacement of radial head in cases of the terrible triad. Josefsson et al.^10^ also concluded that preservation of the radial head and ligament repair are imperative for maintenance of stability in this setting. However radial head replacement is not without its complications (i.e., painful loosening, radioulnar synostosis, dissociation of components, deep infection, capitellar erosion, progressive arthrosis, decreased range of motion, need for re-operation and increased cost). Broberg and Morrey et al.^11^ reported good results in patients with terrible triad injuries treated with radial head excision. We believe the radial head should be preserved if possible, and in fact there were no radial head arthroplasties in this study. Excision of radial head would have been performed only in comminuted fractures. Whenever the radial head is to be excised, strong transosseous ligamentous repair of the medial as well as lateral collateral ligament should be performed, and an ulnohumoral pin should be inserted for a short period.

Heim reviewed AO experience with combined radial and ulnar fractures of the elbow and concluded that reduction of the coronoid fragment is critical to restoration of elbow stability^9^. We believe that elbow stability depends roughly equally on both osseous integrity and soft tissue constraints. The coronoid process, particularly the ulnar fragment is more important because of osseous constraint that it offers against posterior translation of the ulna and attachment of the medial collateral ligament to its base. Since the medial collateral ligament is the primary stabiliser against valgus loading, repairing a coronoid fracture reduces the risk of both valgus and posterior instability. As described above, one useful technique involves using an ACL jig to fix a small avulsed coronoid fragment.

Lateral soft tissue injury was common in the present study. Avulsion of the lateral collateral ligament complex and capsule forming the posterolateral aspect of the distal part of humerus were more common than mid-substance tears and ulnar sided lesions. These structures should be repaired with special attention. We repaired the lateral collateral ligament complex injury by drilling holes in the distal humerus and fixing it with non-absorbable suture. When instability was persistent, medial collateral injury was repaired with a medial incision. We believe that, rather than being detrimental, active elbow motion following surgical repair enhances stability through the recruitment of muscle groups that act as dynamic stabilizers of the elbow.

Our study is limited by the retrospective nature of follow up, its small sample size and by the fact that no patients were treated with radial head prosthesis.

**Table I:**
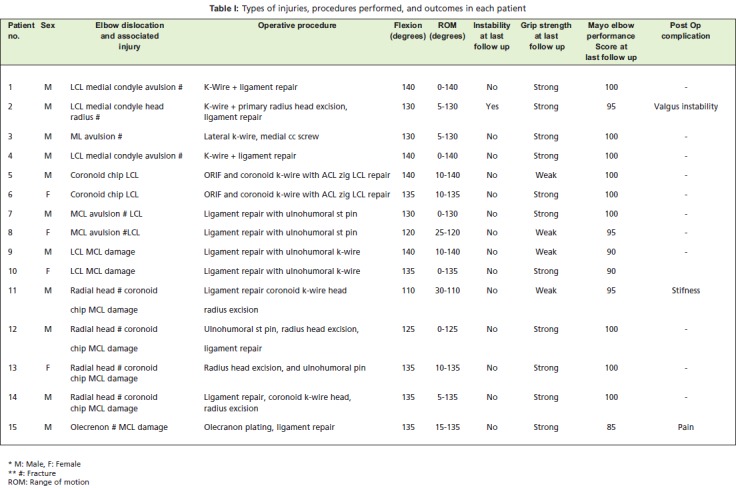
: Types of injuries, procedures performed, and outcomes in each patient

**Figure 3:**
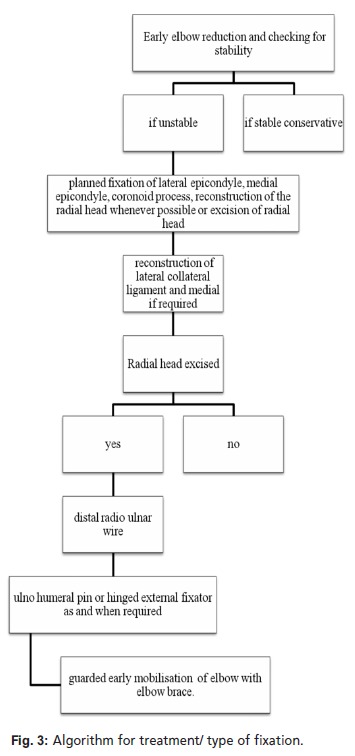
: Algorithm for treatment/ type of fixation

## Conclusion

Following elbow injury, stability of the elbow should be achieved with appropriate attention to the radial head, coronoid, capitellum, lateral collateral ligament, anterior joint capsule and medial collateral ligaments. Planned surgical intervention and early mobilisation improve functional outcome. MEPS scores were good to excellent in younger patients and those with less severe injury. Radial head resection should be used when required to ensure good functional results.

## References

[1] O’Driscoll SW, Jupiter JB, King GJ, Hotchkiss RN, Morrey BF (2001). The unstable elbow. Instr Course Lect..

[2] McKee MD, Schemitsch EH, Sala MJ, O’Driscoll SW (2003). The pathoanatomy of lateral ligamentous disruption in complex elbow
instability. J Shoulder Elbow Surg.

[3] Deutch SR, Jensen SL, Tyrdal S, Olsen BS, Sneppen O (2003). Elbow joint stability following experimental osteoligamentous injury
and reconstruction. J Shoulder Elbow Surg..

[4] Ring DC (2003). Coronoid fracture patterns. Presented at the Annual Orthopaedic Trauma Association Meeting, 2003 October 9-11; Salt
Lake City, Utah..

[5] Ring D, Jupiter JB, Zilberfarb J (2002). Posterior dislocation of the elbow with fractures of the radial head and coronoid. J Bone Joint Surg Am.

[6] Egol KA, Immerman I, Paksima N, Tejwani N, Koval KJ (2007). Fracture-Dislocation of the Elbow Functional Outcome Following
Treatment with a Standardized Protocol.. Bull NYU Hosp Jt.

[7] Pugh DM, Wild LM, Schemitsch EH, King GJ, McKee MD (2004). Standard Surgical Protocol to Treat Elbow Dislocations with Radial
Head and Coronoid Fractures. J Bone Joint Surg Am..

[8] Gill DR, Morrey BF (1998). The Coonrad-Morrey total elbow arthroplasty in patients who have rheumatoid arthritis. A ten to fifteenyear
follow-up study.. J Bone Joint Surg Am.

[9] Heim U (1998). Combined fractures of the radius and the ulna at the elbow level in the adult. Analysis of 120 cases after more than 1
year. Rev Chir Orthop Reparatrice Appar Mot.

[10] Josefsson PO, Gentz CF, Johnell O, Wendeberg B (1989). Dislocations of the elbow and intraarticular fractures. Clin Orthop.

[11] Broberg MA, Morrey BF (1987). Results of treatment of fracture-dislocations of the elbow. Clin Orthop.

[12] Cage DJ, Abrams RA, Callahan JJ, Botte MJ (1995). Soft tissue attachments of the ulnar coronoid process. An anatomic study with
radiographic correlation. Clin Orthop.

[13] Morrey BF, Tanaka S, An KN (1991). Valgus stability of the elbow. A definition of primary and secondary constraints. Clin Orthop.

[14] Boyd HB (1940). Surgical exposure of the ulna and proximal third of the radius through one incision. Surg Gynecol Obstet.

[15] Zagorski JB (1990). Complex fractures about the elbow. Instr Course Lect.

